# Performance evaluation of commercial miRNA expression array platforms

**DOI:** 10.1186/1756-0500-3-80

**Published:** 2010-03-18

**Authors:** Sachin Sah, Matthew N McCall, Deepa Eveleigh, Michael Wilson, Rafael A Irizarry

**Affiliations:** 1 Asuragen Inc., 2150 Woodward St, Suite 100, Austin, TX 78744, USA; 2 Department of Biostatistics, Johns Hopkins Bloomberg School of Public Health, 615 N Wolfe Street, Baltimore, MD 21205, USA

## Abstract

**Background:**

microRNAs (miRNA) are short, endogenous transcripts that negatively regulate the expression of specific mRNA targets. The relative abundance of miRNAs is linked to function *in vivo *and miRNA expression patterns are potentially useful signatures for the development of diagnostic, prognostic and therapeutic biomarkers.

**Finding:**

We compared the performance characteristics of four commercial miRNA array technologies and found that all platforms performed well in separate measures of performance.

**Conclusions:**

The Ambion and Agilent platforms were more accurate, whereas the Illumina and Exiqon platforms were more specific. Furthermore, the data analysis approach had a large impact on the performance, predominantly by improving precision.

## Findings

MicroRNAs (miRNAs) are endogenous, non-coding transcripts that regulate a diverse range of functions, including development, differentiation, growth, apoptosis and metabolism. These 17-24 nucleotide RNA molecules confer specific recognition of target mRNAs and modulate gene expression by acting in conjunction with a set of effector proteins of the RNA interference pathway [1,2]. Through this interaction, miRNAs negatively regulate expression of specific target mRNAs by inhibiting translation, sequestering transcripts in P-bodies [3], or by accelerating mRNA decay as a consequence of rapid deadenylation[4]. Moreover, miRNAs have recently been proposed to activate translation of mRNAs under certain conditions [5].

The relative abundance of miRNAs in cells is thought to be important for miRNAs to exert their regulatory function. For example, titrated expression of both genomic copies of mouse miR-1 is required for normal heart formation and function during embryogenesis [6]. Aberrant miRNA expression contributes to malignancies, tumor progression and metastasis (reviewed in [7]), and miRNA expression profiles can be correlated with disease pathogenesis and prognosis [8,9]. Thus, the performance characteristics of technologies that measure the relative abundance of miRNAs is important for effectively deciphering their functional roles and their potential utility as diagnostic biomarkers.

Microarray technology permits simultaneous expression measurements for hundreds of miRNAs. This technology is already widely used and promises to become a standard tool in the near future. However, a careful assessment of the technology has not yet been performed. This motivated us to evaluate performance attributes of four commercial array platforms for miRNA expression profiling. The miRNA platforms evaluated were Ambion (miRChip; a custom Affymetrix array provided as the DiscovArray™ service through Asuragen,), Agilent (Human miRNA Microarray, v 1.0, GEO accession GPL9081), Exiqon (miRCURY™ LNA Array, v 9.2, GEO accession GPL7724), and Illumina (MicroRNA Expression Profiling Panels, v 1, GEO accession GPL8178). In all cases the sample processing was performed by experienced operators working under standard operating procedures. Samples for three of the four platforms were processed by companies that provide research services on the platform. The study was administered by BIOO Scientific Corporation (Austin, TX) to ensure that the sample identities and purpose of the experiment was blinded. With the exception of the Illumina platform, the laboratory personnel did not know the experiment was part of a performance evaluation. Illumina's Sentrix^® ^Universal-16 BeadChip arrays were used for this study instead of the Sentrix^® ^Array Matrix, which is the manufacturers supported platform for miRNA analysis of the version 1 bead pool.

Seven synthetic miRNAs [Additional file 1] were spiked into a background of 100 ng human placenta total RNA at known input masses ranging from 1 amol to 316 amol in serial 3.16-fold increments. Seven pools of synthetic miRNAs were formulated for spiking according to a 7 × 7 Latin Square design, such that each transcript is spiked in at each concentration (including a zero mass negative control). Endogenous levels of the seven synthetic miRNAs were below the detection threshold when placenta RNA was screened on the Ambion platform. The 100 ng input of total RNA was within the vendors' recommended ranges of inputs. There were substantial differences between platforms in the coverage of miRNAs represented. To eliminate potential probe-content biases in the assessment of precision, we restricted the analysis to 330 human miRNAs represented on all four platforms, representing 45% of the 733 mature human miRNAs registered in the Sanger 10.1 sequence database [10].

Each company provided processed data as part of the standard service using statistical methods produced in house. We refer to these as the default data sets. They are available for download through the NCBI Gene Expression Omnibus (GEO) repository under the following accessions: GSE19248. The Exiqon default data reported the value "NA" (missing values) for 51% of the measurements associated with the spiked-in miRNAs, and 59.1% of the 330. We were, therefore, unable to analyze the Exiqon default data by the methods described, and it was not included in this report. In gene expression microarrays various academic groups have demonstrated that the use of alternative statistical methodology can substantially improve accuracy and precision of expression measurements, relative to ad-hoc procedures developed by the manufacturers of the technology [11]. We therefore also used the raw probe-level data from all companies, with the exception of Agilent. The Agilent miRNA platform typically interrogates repeated measurements of two probes per miRNA that are summarized using a proprietary algorithm. Therefore, Agilent does not recommend using raw probe-level data for data analysis or normalization. We compared two alternative approaches to background correction to the default: no-background correction and exponential-normal convolution [11]. We also compared quantile normalization [12] to the default normalization method for each platform. We refer to the processed data (in log_2 _scale) as *expression values*. We found that no-background correction and quantile normalization clearly outperformed other approaches, so we used these methods to compare platform performance. For Agilent we used the default dataset according to the vendor's recommendations. Figures using the default dataset for all platforms are included as Additional files 2, 3, 4.

We assessed specificity and sensitivity in a way that can be easily related to practical performance. The use of the same placental total RNA as background material in each hybridization permitted us to assess specificity. Spike-in experiments have been used extensively to assess gene expression technologies as they provide a sensible way of measuring sensitivity [13,14]. However, misleading conclusions can be drawn from experiments with unusually high expression measurements for the spike-in concentrations that presumably do not represent the nominal concentrations of the background RNA [15]. For this reason, we carefully calibrated our spike-in material to assure that the distribution of observed expression for the spike-in transcripts reflects the distributions seen in typical experiments. Additional file 2 shows the typical distribution of expression values for the background RNA for the four studied data sets. The tick marks on the x-axis represent the average expression at each reported spike-in level. This figure illustrates that the spike-in transcripts resulted in expression measurements similar to the background RNA transcripts.

**Figure 1:**
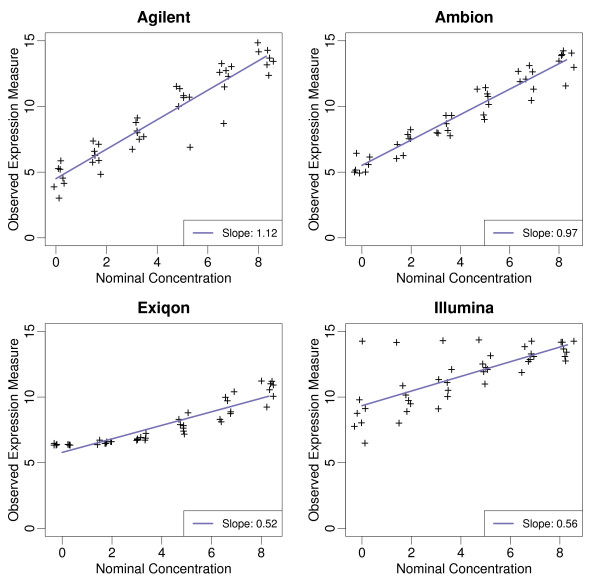
**Observed versus nominal values**: For each of the four platforms, expression values of spiked miRNAs are plotted against the log (base_2_) of the reported nominal concentration. The regression line and slope are shown.

We adapted statistical assessments that have been successfully implemented for gene expression arrays [16]. We start with a basic assessment of accuracy: the *signal detection slope *[16]. Microarray expression values intend to measure the abundance of sample RNA. Therefore we expect that a doubling of nominal concentration would result in a doubling of observed intensities. In other words, on the log (base_2_) scale, the slope from the regression of expression on nominal concentration can be interpreted as the expected observed difference when the true difference is a fold change of 2. Thus, an optimal result is a slope of one and values higher and lower than one are associated with over and under estimation, respectively. Figure 1 demonstrates that Ambion performed best in the assessment of accuracy. The lower accuracy of the Exiqon platform can be attributed to poor dose-responsiveness at the low-mass inputs. This apparent reduced sensitivity at low mass input is consistent with a relatively high proportion of non-detected probes (59.1%) that were reported in the default data set. The expression signals corresponding to one spiked miRNA on the Illumina array were high and correlated poorly with the input doses (See Figure 1). Removing this aberrant probe produced a relative accuracy slope of 0.65, in contrast to 0.56 reported in Table 1. The inconsistent performance of one probe corresponding to one of the spiked-in transcripts may indicate a selectivity bias with the underlying probe design or labeling assay. This possibility was not addressed with this experimental design.

**Table 1:** Assessment results:

Platform	Preprocessing	Slope (SD)	SD	99%	SNR	TOP	NA%
Illumina	QN	0.56 (1.02)	0.15	0.88	3.73	0.38	0

Exiqon	QN	0.52 (0.75)	0.14	0.98	3.71	0.27	0

Ambion	QN	0.97 (0.75)	0.27	1.91	3.59	0.17	0

Agilent	Default	1.12 (0.66)	0.32	1.91	3.50	0.11	14.63

Illumina	BGC & QN	0.61 (1.11)	0.18	1.45	3.39	0.22	0

Illumina	Default	0.60 (1.15)	0.24	2.55	2.5	0.04	4.89

Ambion	BGC & QN	1.20 (1.55)	0.55	4.02	2.18	0.03	0

Exiqon	BGC & QN	1.02 (1.02)	0.47	2.97	2.17	0.03	0

Ambion	Default	1.12 (1.34)	0.55	3.92	2.04	0.02	0

For each platform, we report summary assessments for accuracy, precision, and overall performance. The first column shows the signal detection slope which can be interpreted as the expected observed difference when the true difference is a fold change of 2. In parenthesis is the standard deviation of the log-ratios associated with non-zero nominal log-ratios. The second column shows the standard deviation (SD) of the log-ratio null distribution. The SD can be interpreted as the expected range of observed log-ratios for genes that are not differentially expressed. The third column shows the 99th percentile of this null distribution. It can be interpreted as the expected minimum value that the top 1% of non-differentially expressed miRNA will reach. The fourth column shows the ratio of the values in column 1 and column 2. It is a rough measure of signal to noise ratio. The fifth column shows the probability that, when comparing two samples, a gene with true log fold change of 2 will appear in a list of the top 1% genes with the highest log-ratios. The sixth column shows the percentage of negative values in the default data set.

**Figure 2:**
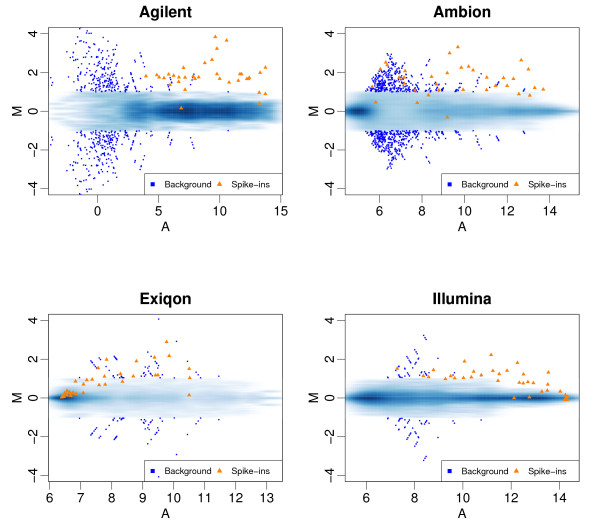
**MA plots**: For each platform, we performed all pair-wise comparisons of the seven arrays. From each comparison we computed the log-ratio (M) and average expression value (A) for each miRNA feature. These plots show M plotted against A. To avoid drawing hundreds of points on top of each other we use a *smooth scatter *plot which shows the distribution of these points: dark and light shades of blue show high and low frequency of points, respectively. The points associated with spike-in transcripts with nominal fold changes of 3.16 are shown as orange triangles. Points associated with larger nominal fold changes are not shown since they were very easy to detect for all platforms. Points not associated with the spike-in transcripts (should have M = 0) that achieved fold changes above 2 are shown as large blue squares.

Specificity is another important feature of array-based platform performance. Because the majority of microarray studies rely on relative measures (e.g. fold change) as opposed to absolute ones, we focused on the precision of the basic unit of relative expression: log-ratios. We adapted the precision assessment of Cope et al. [16] that focused on the variability of log-ratios generated by comparisons expected to produce log-ratios of 0. This was achieved by using comparisons within the background RNA. We refer to this group of comparisons as the *Null *set. The standard deviation (SD) of these log-ratios serves as a basic assessment of precision and has a useful interpretation: it is the expected range of observed log-ratios for genes that are not differentially expressed. In gene expression arrays, specificity performance has been shown to vary with nominal concentrations [17]. We therefore plotted the log-ratios against the average expression value for each comparison or MA-plots. Figure 2 combines the results from all pair-wise comparisons of the seven arrays and includes the values obtained for the transcripts spiked in with nominal log-ratios of 1.66, the smallest nominal value produced by our design. To avoid plotting thousands of points on top of each other we use a two dimensional density plot: color intensity represents the frequency of observations at each point (darker = higher frequency). Fold-change values from null set larger than 2 are considered false positives and are shown with blue squares. The results for the spike-in transcripts are shown with orange triangles. A platform that performs well should show clear separation between the null set and the spiked-in set: the orange triangles should separate from the blue regions and we should see no blue squares. Figure 2 highlights two important findings: 1) Precision depends on concentration with higher variability observed for low concentrations. 2) Illumina and Exiqon, which had the worst accuracy, have the best precision. The overall separation was slightly better for the methods with better precision. The MA plots for the default data analysis [Additional file 3] demonstrate increased variance compared to no-background correction/quantile normalization. The gains in accuracy are not enough to overcome the reduced ability to discriminate signal from noise.

Note that in that in Figure 2, many dark blue dots were observed on each platform. This was expected given the documented problem of cross-hybridization. Because a platform with larger SD and small outliers might be preferable to one with a smaller SD but large outliers we included the 99^th ^percentile of the null distribution as a second summary assessment of specificity. Note that for this analysis 3.3 is the expected 1% value for the 330 human mature miRNAs common to all platforms. However, the number of array features will certainly increase in the near future: the number of false positives (in the top 1%) will increase proportionally.

Precision and accuracy assessments, considered independently, have limited practical use. However, the summary statistics described above can be easily combined to answer many practical questions when posed in a statistical context. As an example, we computed the chance that, when comparing two samples, a gene with true log_2 _fold change, Δ = 1, will appear in a list of the top 1% (highest log-ratios). This summary statistic, as well as the accuracy and precision summaries described above are shown in Table 1. Note that Table 1 includes results for all the data analysis approaches we considered.

We have described an assessment procedure for microRNA microarray data based on a carefully designed spike-in experiments. Strengths and weaknesses were revealed for each platform. Ambion and Agilent were more accurate, while, Illumina and Exiqon were more specific. Strikingly, the data processing methods had a more profound impact on the performance than were observed for differences between platforms. The introduction of background correction adjustment to the raw data was detrimental to specificity, inferring that background correction was the likely cause of lower performance for the three default data sets. The practical implication is that false positive fold changes are most likely to be detected at lower expression signals from default data, and may be reduced by eliminating the background correction from the raw data.

We considered quantile normalization to be the best approach among multiple options for this study design because the distribution of the background RNA is identical across the project. For projects where the miRNA fraction of total RNA may be variable across different samples in the project, another normalization method may be more appropriate.

The experimental design did not include measurements of day-to-day or site-to-site variability to evaluate platform robustness, so we were not able to draw direct conclusions as to whether these platforms might have performed differently under different circumstances. Reproducibility testing of the Agilent, Ambion and Illumina platforms beyond the scope of this study suggested that the performance reported here is within the expected day-to-day variability (not shown).

Both Ambion and Agilent demonstrated good accuracy across the range tested but with less precision than the other two platforms. Agilent performed the best when only the default data set was evaluated for each platform. Considering that we adhered to Agilent's guidance to use the default data, further analysis is required to determine whether excluding the background adjustment or including a global normalization method can improve the performance of the Agilent array.

## Competing interests

The authors SS and DE are employees of Asuragen. Asuragen provides a commercial service on the DiscovArray™ and Agilent platforms and funded the experiments described in this study. The authors MM, MW and RI declare that they have no competing financial interests.

## Authors' contributions

MW and RI designed the research. SS and DE carried out the experiment MM carried out the data analysis. MM, MW, and RI wrote the manuscript. All authors read and approved the final manuscript.

## Supplementary Material

Additional file 1 **Supplementary Table S1**. Spike-in sequence Click here for file

Additional file 2 **Supplementary Figure S1 - Empirical densities**: These plots depict the empirical density of the average (across arrays) expression values for the background RNA, including quantile normalized raw data (A) and default data (B). The tick marks on the x-axis show the average expression at each nominal spike concentration. Click here for file

Additional file 3 **Supplementary Figure S2**. As Figure 2 but using the default preprocessing procedures. Click here for file

Additional file 4 **Supplementary Figure S3**. As Figure 1 but using the default preprocessing procedures. Click here for file

## References

[B1] Bartel DP MicroRNAs: genomics, biogenesis, mechanism, and function Cell 2004 116 281 297 10.1016/S0092-8674(04)00045-5 14744438

[B2] Gregory R Chendrimada T Cooch N Shiekhattar R "Human RISC couples microRNA biogenesis and posttranscriptional gene silencing" Cell 2005 123 4 631 40 10.1016/j.cell.2005.10.022 16271387

[B3] Liu J Valencia-Sanchez MA Hannon GJ Parker R MicroRNA-dependent localization of targeted mRNAs to mammalian P-bodies Nat Cell Biol 2005 7 719 23 10.1038/ncb1274 15937477 PMC1855297

[B4] Wu L Fan J Belasco JG MicroRNAs direct rapid deadenylation of mRNA Proc Natl Acad Sci USA 2006 103 4034 9 10.1073/pnas.0510928103 16495412 PMC1449641

[B5] Vasudevan S Tong Y Steitz JA Switching from Repression to Activation: MicroRNAs Can Up-Regulate Translation Science 2007 318 1931 1934 10.1126/science.1149460 18048652

[B6] Zhao Y Dysregulation of cardiogenesis, cardiac conduction, and cell cycle in mice lacking miRNA-1-2 Cell 2007 129 303 17 10.1016/j.cell.2007.03.030 17397913

[B7] Esquela-Kerscher A Slack FJ Oncomirs - microRNAs with a role in cancer Nat Rev Cancer 2006 6 259 269 10.1038/nrc1840 16557279

[B8] Schetter AJ MicroRNA expression profiles associated with prognosis and therapeutic outcome in colon adenocarcinoma Jama 2008 299 425 36 10.1001/jama.299.4.425 18230780 PMC2614237

[B9] Yu SL MicroRNA signature predicts survival and relapse in lung cancer Cancer Cell 2008 13 48 57 10.1016/j.ccr.2007.12.008 18167339

[B10] Griffiths-Jones S miRBase: the microRNA sequence database Methods Mol Biol 2006 342 129 38 16957372 10.1385/1-59745-123-1:129

[B11] Yang YH Normalization for cDNA microarray data: a robust composite method addressing single and multiple slide systematic variation Nucleic Acids Res 2002 30 e15 10.1093/nar/30.4.e15 11842121 PMC100354

[B12] Bolstad BM Irizarry RA Astrand M Speed TP A comparison of normalization methods for high density oligonucleotide array data based on variance and bias Bioinformatics 2003 19 185 93 10.1093/bioinformatics/19.2.185 12538238

[B13] Lockhart DJ Expression monitoring by hybridization to high-density oligonucleotide arrays Nat Biotechnol 1996 14 1675 80 10.1038/nbt1296-1675 9634850

[B14] Hughes TR Expression profiling using microarrays fabricated by an ink-jet oligonucleotide synthesizer Nat Biotechnol 2001 19 342 7 10.1038/86730 11283592

[B15] Irizarry RA Cope LM Wu Z Feature-level exploration of a published Affymetrix GeneChip control dataset Genome Biol 2006 7 404 10.1186/gb-2006-7-8-404 16953902 PMC1779590

[B16] Cope LM Irizarry RA Jaffee HA Wu Z Speed TP A benchmark for Affymetrix GeneChip expression measures Bioinformatics 2004 20 323 31 10.1093/bioinformatics/btg410 14960458

[B17] Irizarry RA Wu Z Jaffee HA Comparison of Affymetrix GeneChip expression measures Bioinformatics 2006 22 789 94 10.1093/bioinformatics/btk046 16410320

