# Supplementary material for: Performance evaluation of commercial miRNA expression array platforms
Source: BMC Res Notes. 2010 Mar 18;3:80. doi: 10.1186/1756-0500-3-80 (PMC2853548; doi:10.1186/1756-0500-3-80)
Supplement: Additional file 1 — Supplementary Table S1. Spike-in sequence [file 1756-0500-3-80-S1.DOCX]

| Spiked_miRNA_ID | Sequence |
| --- | --- |
| hsa-miR-147 | GUGUGUGGAAAUGCUUCUGC |
| hsa-miR-211 | UUCCCUUUGUCAUCCUUCGCCU |
| hsa-miR-219 | UGAUUGUCCAAACGCAAUUCU |
| hsa-miR-302d | UAAGUGCUUCCAUGUUUGAGUGU |
| hsa-miR-338 | UCCAGCAUCAGUGAUUUUGUUGA |
| hsa-miR-383 | AGAUCAGAAGGUGAUUGUGGCU |
| hsa-miR-429 | UAAUACUGUCUGGUAAAACCGU |
