# Supplementary figures and images for: Performance evaluation of commercial miRNA expression array platforms
Source: BMC Res Notes. 2010 Mar 18;3:80. doi: 10.1186/1756-0500-3-80 (PMC2853548; doi:10.1186/1756-0500-3-80)

Supplemental Figure 1A

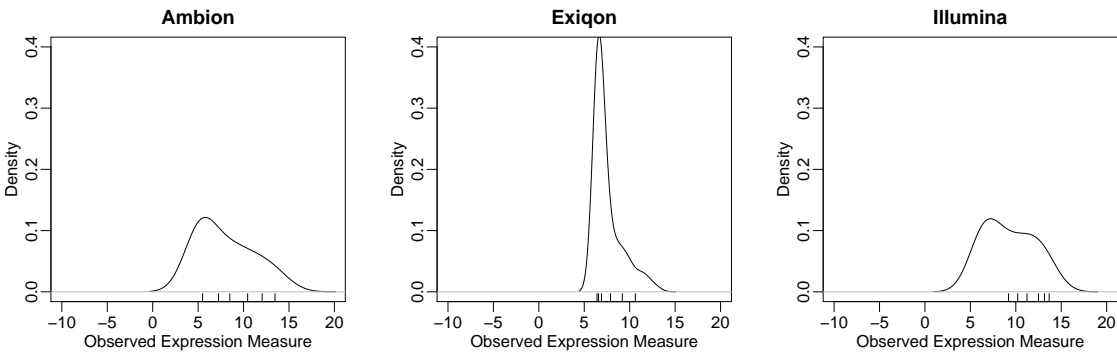

Supplemental Figure 1B

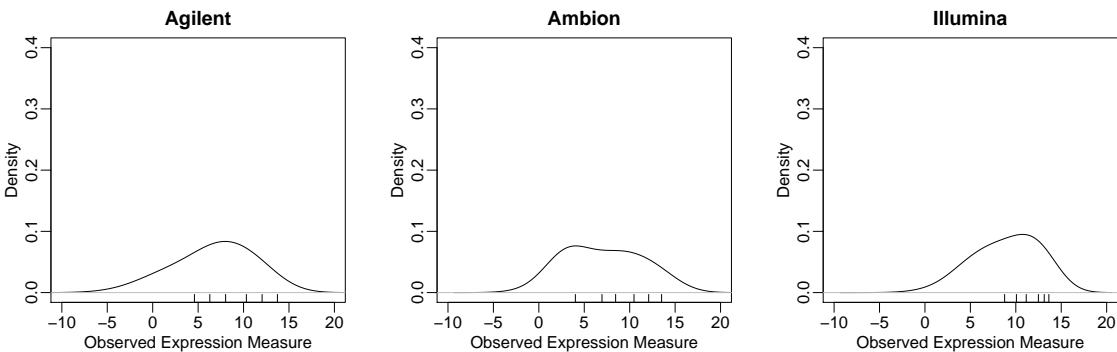

Supplement: Additional file 2 — Supplementary Figure S1 - Empirical densities: These plots depict the empirical density of the average (across arrays) expression values for the background RNA, including quantile normalized raw data (A) and default data (B). The tick marks on the x-axis show the average expression at each nominal spike concentration. [file 1756-0500-3-80-S2.PDF]

Supplemental Figure 2

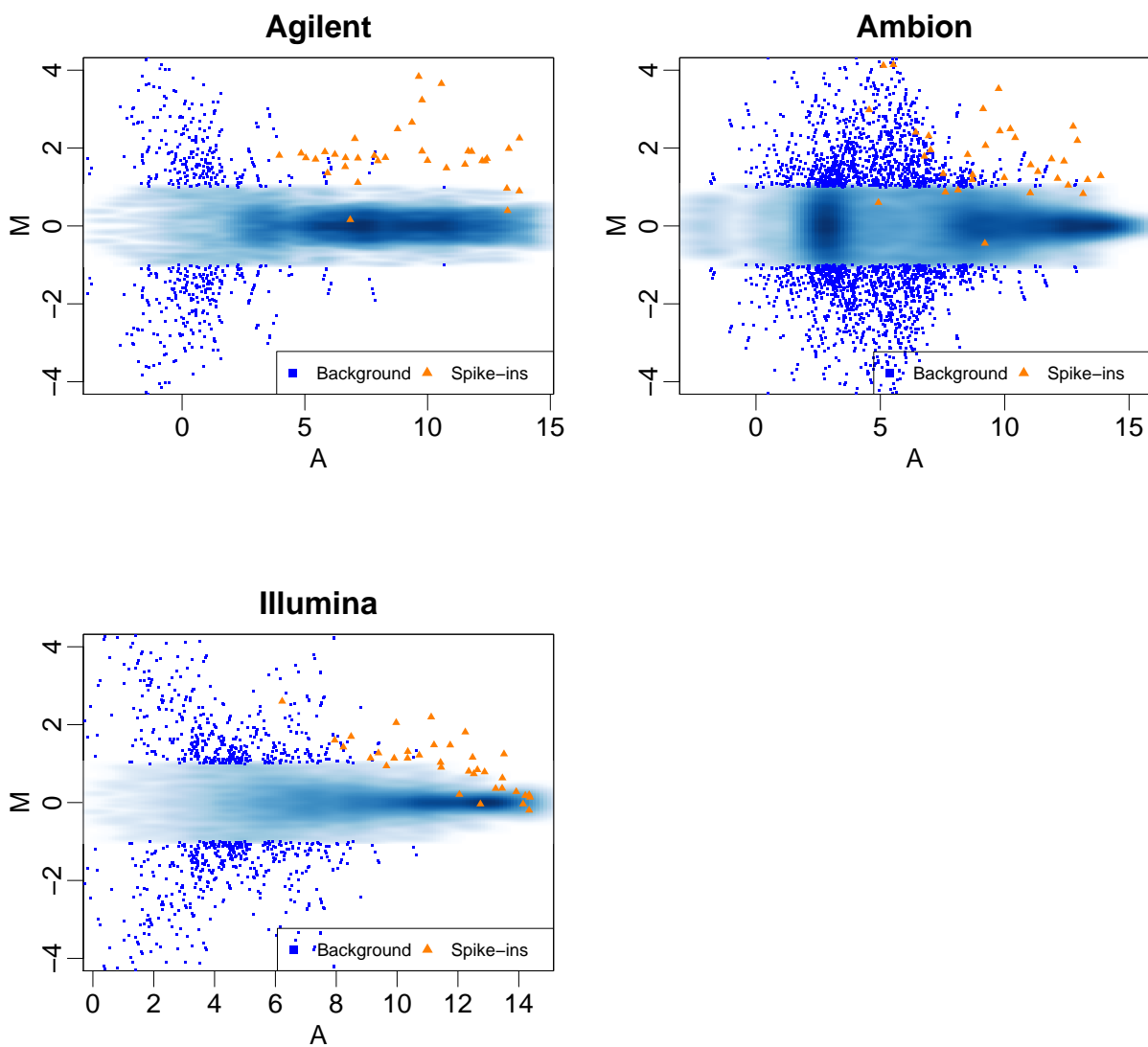

Supplement: Additional file 3 — Supplementary Figure S2. As Figure 2 but using the default preprocessing procedures. [file 1756-0500-3-80-S3.PDF]

Supplemental Figure 3

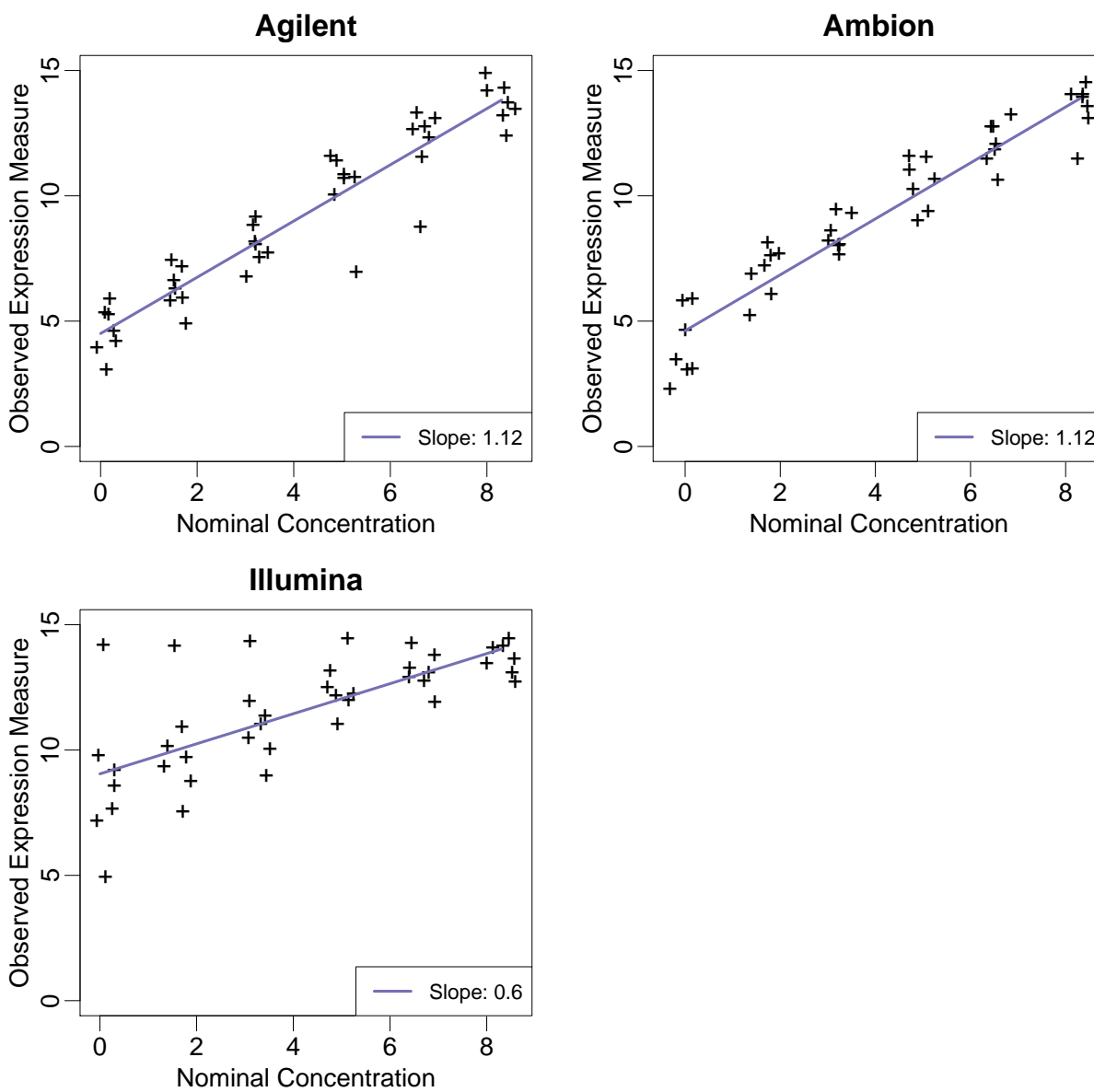

Supplement: Additional file 4 — Supplementary Figure S3. As Figure 1 but using the default preprocessing procedures. [file 1756-0500-3-80-S4.PDF]
